# Accessing the nursing behaviour of Moso bamboo (*Phyllostachys edilus*) on carbohydrates dynamics and photosystems

**DOI:** 10.1038/s41598-020-57643-1

**Published:** 2020-01-23

**Authors:** Shitephen Wang, Tsai-Huei Chen, En-U Liu, Chiung-Pin Liu

**Affiliations:** 1 Kyoto University, Graduate School of Agriculture, Kyoto, 606-0096 Japan; 2 Taiwan Forest Research Institute, Silviculture Division, Taipei, 10066 Taiwan; 3 Taiwan Forest Research Institute, Forestry Economics Division, Taipei, 10066 Taiwan; 4 National Chung Hsing University, Department of Forestry, Taichung, 40227 Taiwan

**Keywords:** Plant physiology, Behavioural ecology

## Abstract

Nursing behaviour, also known as breastfeeding behaviour, is the feeding of juvenile individuals with nutrients or proteins from matures especially in mammals. As a hypothetical phenomenon in bamboo forests, mature bamboos have transferred photoassimilates to young bamboos for recovering and rebuilding their photosystems especially in winter. This process is accompanied by changes in the ability of photosystems and the mass fraction of non-structural carbohydrates (NSCs), structural carbohydrates (SCs), and lignin. We analysed carbohydrates and chlorophyll fluorescence to compare the physiological traits in mature (age 2, 3, 4) and immature (age 1) Moso bamboos (*Phyllostachys edilus*) during a year using the Portable Chlorophyll Fluorometer (PCF) and the Liquid Chromatographic (LC) method. The results showed that the mass fraction of total soluble carbohydrates (TSCs) and starch in the bottom of bamboo at age 1 was higher than other parts and ages in spring, whereas the mass fraction of TSCs, starch, and sucrose at age 3 was higher than other parts and ages in winter. The Fv/Fm, an indicator to reveal photosystems were functional or not, at age 1 dramatically dropped when the cold current attacked first time in October, and then quickly recovered in November. Our findings indicate that mature bamboos very possibly provide carbohydrates to immature bamboos and help them rebuild their photosystems when a bamboo forest resists cold stress.

## Introduction

Moso bamboo (*Phyllostachys edilus*) is a kind of broadly distributed bamboo species with its leptomorph rhizome systems in east Asia^1–4^. Because it has leptomorph rhizome systems, some studies assume that mature bamboos perhaps provide nutrients and carbohydrates to bamboo shoot^5^ and juvenile bamboos^1,3,6^. This phenomenon may be called a kind of “nursing behaviour” which helps juvenile individuals (e.g. bamboo shoots and young bamboos) to quick grow or recover photosynthetic function after extreme weather events in bamboo forests.

For clarifying the phenomenon of carbohydrate-dependent in new born bamboos^7^ and the speculation of which carbohydrates and nutrients needed for the construction of new bamboos are provided by other attached mature bamboos via underground (also known as belowground) rhizomes^8^, a research team proved the hypothesis that the non-structural carbohydrates (NSCs) needed for the structure carbohydrates (SCs) and metabolism of bamboo shoots during the fast growth period (FGP) probably are provided by attached mature bamboos via belowground rhizomes^5^.

NSCs and some parts of SCs are a kind of format for storing energy in plants and Moso bamboos. As the important compounds from plant photosynthesis, carbohydrates can be synthesised into NSCs and SCs according to their purposes which depend on different environments and conditions^9,10^. SCs, including cellulose, hemicelluloses, lignin, and pectin, are used for the structural growth in plants^11^. The NSCs pool is the sum of soluble sugars and starches, and NSCs can be remobilised for multiple usages and play an important role in plant defences, germination, growth, reproduction and survivorship under stresses^12^. NSCs probably can provide a temporary source of carbon when current photosynthesis cannot meet the immediate carbon demands of bamboo^13^.

**Figure 1:**
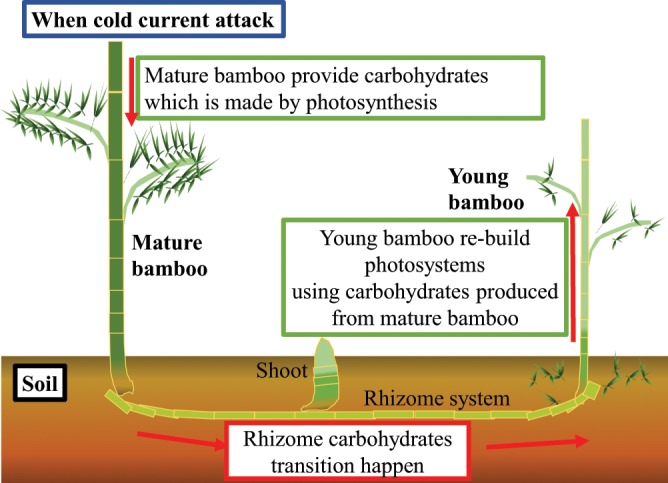
Carbohydrates transition pathway among young, mature bamboo, and rhizome when Moso bamboo forest when the cold current attack. This figure was designed and obtained by Shitephen Wang using Adobe Photoshop cc 2016 & Adobe Illustrator cc 2016 (Adobe Systems Software Ireland Ltd).

On the other hands, however, previous studies rarely have been conducted to measure the carbohydrates [such as NSCs^5^ and SCs] dynamics and especially the photosynthetic ability in bamboo forests. We aimed to illustrate one of the nursing behaviours that mature bamboos provide carbohydrates to immature bamboos and help them to recover their photosystems after extreme weather events probably via underground rhizomes, and then reduce the mortality rate of young individuals (Fig. 1).

## Results

### Non-structural carbohydrates (NSCs) in different seasons and bamboo ages

**Figure 2:**
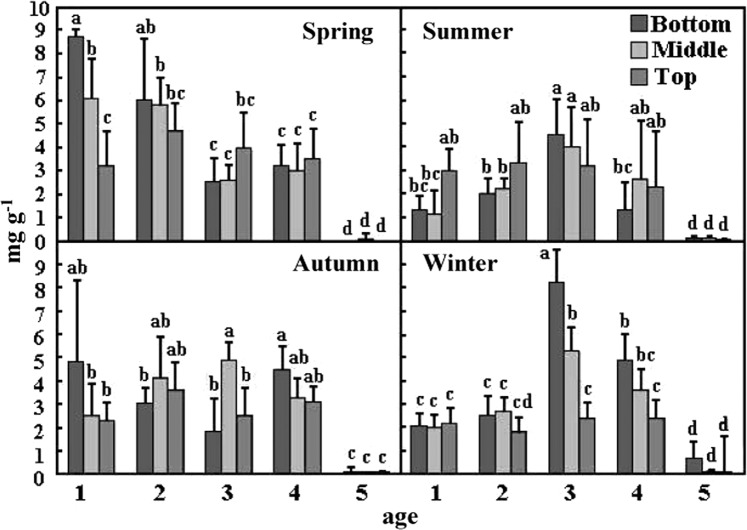
The mass fraction of total soluble carbohydrate of Moso bamboo in different ages during the year. Values with different letters are significantly different at 5% significant level by LSD.

**Figure 3:**
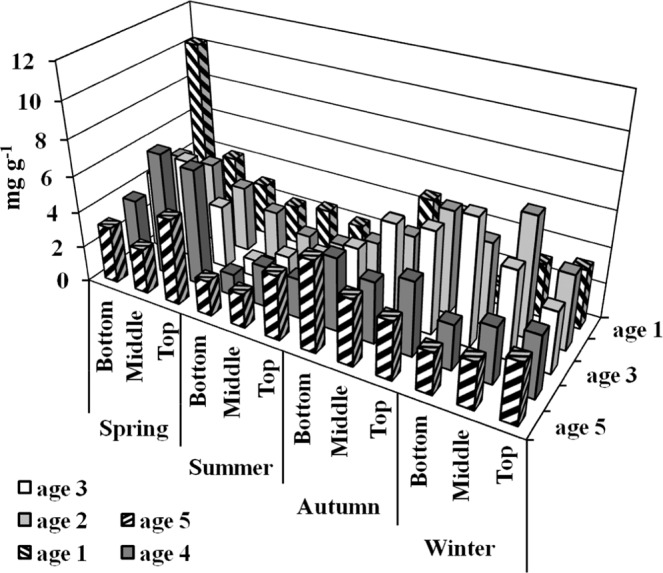
The mass fraction of starch of Moso bamboo in different ages during the year.

NSCs, including soluble carbohydrates and starch. Among them, soluble carbohydrates are mostly composed of glucose, fructose, and sucrose, while starch is polymerised from glucose. Total soluble carbohydrates (TSCs) in different seasons and bamboo ages show that analysis of the TSCs of Moso bamboos in different seasons is shown in Fig. 2. The TSCs in the young bamboo (age 1) bottom in spring and the mature bamboo (age 3) bottom in winter is significantly higher than that in other seasons and bamboo ages. In the old bamboo (age ≥ 5), all the parts of TSCs are significantly lower than mature and young bamboos in all seasons. The analysis of starches in different seasons and bamboo ages shows that bamboos’ mass fraction of starch in summer is higher than in other seasons. In particular, young bamboos’ bottom (age 1) is the highest mass fraction of starch in spring (Fig. 3).

**Figure 4:**
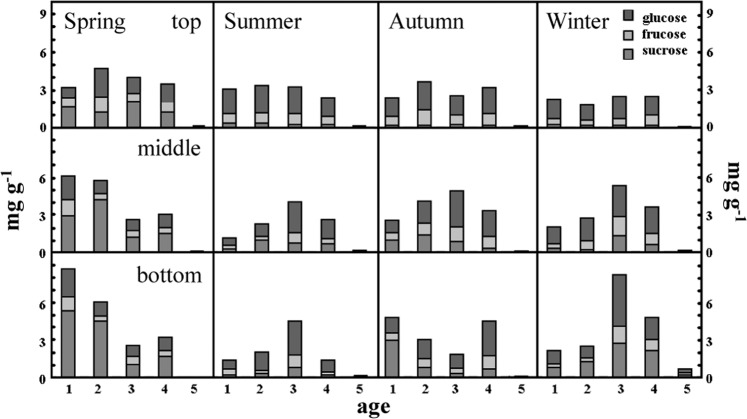
The mass fraction of different soluble carbohydrates of Moso bamboo in different ages during the year.

**Figure 5:**
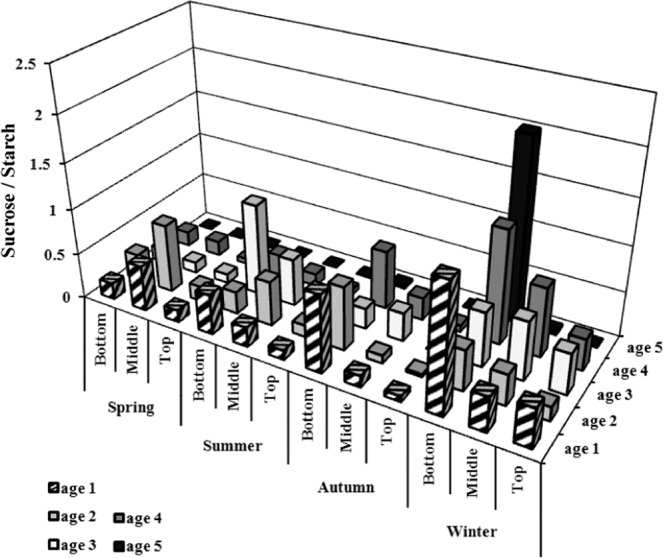
The sucrose/starch ratio of Moso bamboo in different ages during one year.

The mass fraction of TSCs is highest in the bottom of young bamboos (age 1) in spring and lowest in old bamboos (≥age 5) in all seasons. In spring, the mass fraction of sucrose is higher than that of glucose and fructose in different ages of bamboo. Among them, the mass fraction of glucose in the bottom of young bamboo (age 1) is relatively higher than other ages (age 2, 3, 4, and ≥5) (Fig. 4). In summer, the mass fraction of TSCs is relatively high in the bottom of age 3 bamboo and extremely low in old bamboos (age ≥ 5), and that of glucose is higher than sucrose and fructose in the bottom of age 3 bamboos (Fig. 4). Furthermore, the bottom of age 3 bamboo has the highest sucrose/starch ratio (Fig. 5). The mass fraction of TSCs in the middle of age 3 and in the bottom of age 1 and age 4 is higher than other ages content in autumn, while that of the old bamboo (age ≥ 5) is lowest in the bamboo forest. Mature bamboos (age 2, 3, and 4) had a higher mass fraction of glucose in the autumn as well, while young bamboos (age 1) had a higher mass fraction of sucrose and that of fructose had no significant difference between young and mature bamboos (Fig. 4). Further, the sucrose/starch ratio at the bottom of age 1, 2, and 4 bamboos are relatively higher than other parts and ages (Fig. 5). In winter, the mass fraction of TSCs is highest in age 3 bamboos and lowest in old bamboos (age ≥ 5). A high-level mass fraction of sucrose is in the bottom of age 3 bamboos, whereas a low-level of that in the top of age 3 bamboos. And age 4 bamboos have a similar situation with age 3 bamboos (Fig. 4). The sucrose/starch ratio in the bottom of all bamboos is higher than other seasons except for age 3 bamboos (Fig. 5).

### Ability of photosynthetic system in different seasons and bamboo ages

**Figure 6:**
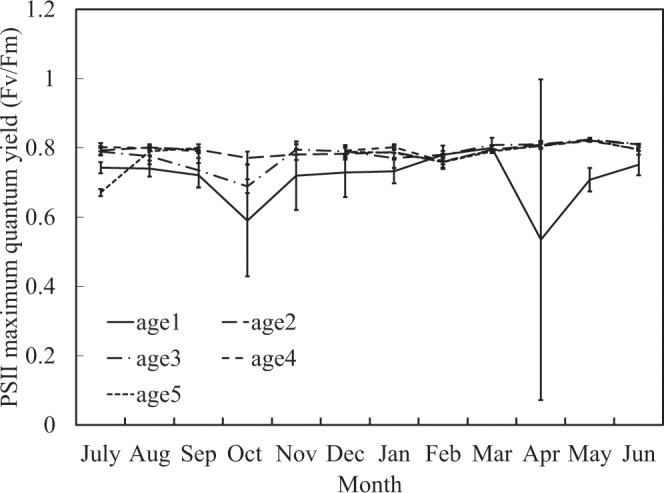
PSII maximum quantum yield (Fv/Fm) in different ages during one year.

**Figure 7:**
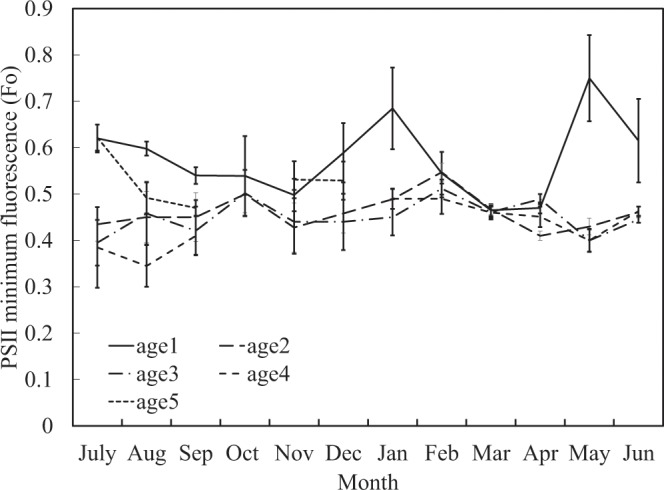
PSII minimum fluorescence (Fo) in different ages during one year.

**Figure 8:**
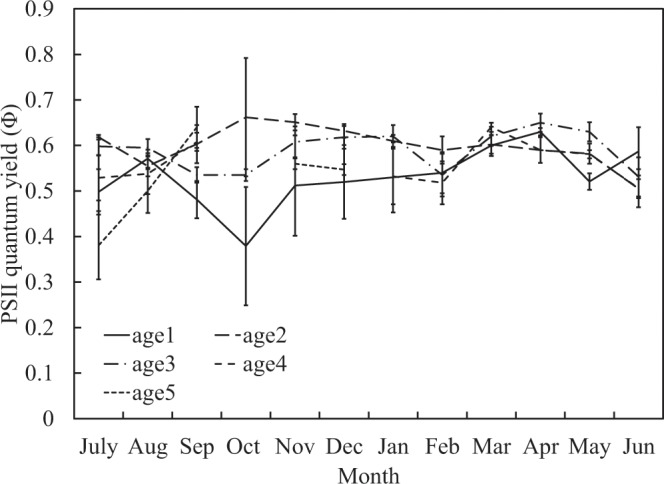
PSII quantum yield (Φ) in different ages during one year.

**Figure 9:**
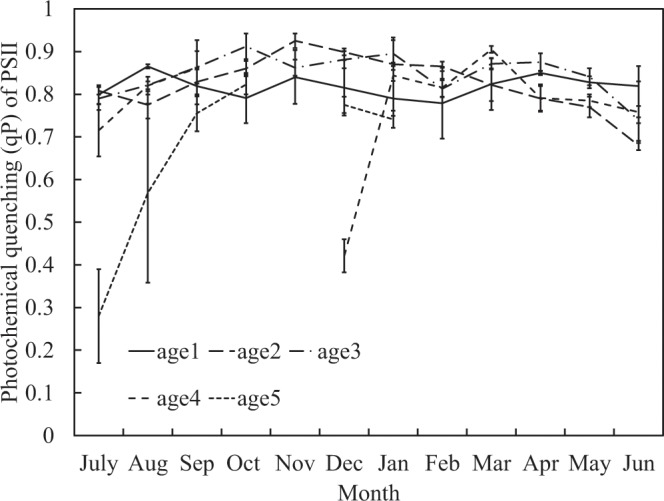
Photochemical quenching (qP) of PSII in different ages during one year.

In order to assess the ability of Moso bamboos’ photosynthetic systems, we determine the PSII maximum quantum yield (Fv/Fm), PSII minimum fluorescence (Fo), PSII quantum yield (Φ), and photochemical quenching (qP) of PS II on study site. Fv/Fm is generally between 0.7 and 0.8; however, in April and October, young bamboos’ (age 1) leaves have a significant downward trend (Fig. 6). Furthermore, Fo is between 0.4 and 0.55, but young bamboo in leaves has a significant rise in winter and May (Fig. 7). Φ is generally about 0.5–0.7; however, the old bamboo (age ≥ 5) in July and young bamboo (age 1) in autumn and winter (especially in October when the first cold current attacked) are significantly decreasing (Fig. 8). The qP value is normally between 0.7 and 0.9. When the age 4 bamboos grow new leaves in November and old bamboos leaves in July, the qP value is significantly lower than other ages (Fig. 9).

### Structural carbohydrates (SCs) in different seasons and bamboo ages

Overall, the average mass fraction of lignin, xylan, arabinan, mannan, glucan, and galactan in this study was at 30.5%, 23.9%, 1.7%, 0.3%, 42.0%, and 0.7% (305, 239, 17, 3, 420 and 7 g kg^−1^), respectively (Table 1). The lignocellulosic ethanol index (LEI) is about 2.2 and is higher in spring than in other seasons. The LEI of age 4 in spring and that of age 1 in summer are as high as 3.0, whereas the LEI of age 4 in autumn and that of old bamboos (age ≥ 5) in summer, autumn, and winter are lower than other ages in other seasons. The average torrefaction index (TI) is at 1.2, and is highest in autumn. Further, the TI of old bamboos (age ≥ 5) in summer, autumn, and winter is as high as 1.5, whereas that of young bamboos (age 1) in summer is the lowest one in other ages and seasons (Table 1).

**Table 1:** The mass fraction of structural carbohydrate in different ages during the year.

Season	age	lignin	xlyan	arabinan	mannan	glucan	galactan	lignocellulosic ethanol index	torrefaction index
		(g kg^−1^ ± SD)							
Spring	1	304 ± 44	252 ± 12	19 ± 5	3 ± 1	406 ± 42	7 ± 5	2.2 ± 0.5	1.1 ± 0.1
	2	288 ± 44	252 ± 9	18 ± 4	3 ± 1	423 ± 42	6 ± 3	2.4 ± 0.5	1.0 ± 0.1
	3	274 ± 24	254 ± 1	18 ± 3	7 ± 4	432 ± 24	6 ± 1	2.5 ± 0.3	1.0 ± 0.1
	4	247 ± 44	247 ± 8.8	18 ± 5	7 ± 3	464 ± 47	8 ± 4	3.0 ± 0.7	0.9 ± 0.2
	5	292 ± 48	24.0 ± 10	18 ± 3	1 ± 0.5	428 ± 30	10 ± 6	2.3 ± 0.6	1.1 ± 0.1
Summer	1	160 ± 32	325 ± 17	15 ± 9	11 ± 4	289 ± 25	4 ± 4	3.0 ± 0.5	0.5 ± 0.07
	2	304 ± 23	261 ± 10	15 ± 2	4 ± 1	397 ± 16	9 ± 6	2.2 ± 0.2	1.1 ± 0.04
	3	311 ± 23	252 ± 8	15 ± 3	2 ± 0.3	406 ± 14	4 ± 1	2.1 ± 0.2	1.1 ± 0.04
	4	306 ± 5	244 ± 1	17 ± 4	3 ± 0.8	413 ± 13	6 ± 3	2.1 ± 0.1	1.1 ± 0.04
	5	353 ± 08	206 ± 4	15 ± 5	1 ± 0.0.8	406 ± 10	8 ± 3	1.7 ± 0.05	1.5 ± 0.03
Autumn	1	310 ± 21	218 ± 2	15 ± 5	1 ± 0.6	441 ± 31	5 ± 3	2.1 ± 0.2	1.3 ± 0.1
	2	315 ± 19	214 ± 11	14 ± 5	1 ± 0.4	441 ± 30	6 ± 4	2.1 ± 0.2	1.3 ± 0.1
	3	318 ± 04	215 ± 10	15 ± 4	1 ± 0.3	436 ± 10	6 ± 2	2.0 ± 0.03	1.3 ± 0.03
	4	329 ± 10	205 ± 3	15 ± 4	1 ± 0.3	432 ± 19	8 ± 3	1.9 ± 0.1	1.4 ± 0.1
	5	360 ± 19	178 ± 12	16 ± 4	1 ± 0.3	428 ± 28	8 ± 2	1.7 ± 0.1	1.8 ± 0.1
Winter	1	301 ± 45	264 ± 16	21 ± 2	4 ± 2.5	390 ± 31	10 ± 3	2.2 ± 0.5	1.0 ± 0.1
	2	289 ± 18	261 ± 4	17 ± 3	3 ± 1	412 ± 18	8 ± 2	2.3 ± 0.2	1.0 ± 0.1
	3	298 ± 18	259 ± 2	17 ± 3	2 ± 1	407 ± 24	7 ± 2	2.2 ± 0.2	1.0 ± 0.1
	4	288 ± 14	260 ± 18	16 ± 1	2 ± 1	419 ± 30	4 ± 1	2.4 ± 0.2	1.0 ± 0.1
	5	355 ± 17	209 ± 8	12 ± 3	1 ± 0.5	408 ± 13	4 ± 3	1.7 ± 0.1	1.6 ± 0.03
Average		300 ± 40	241 ± 20	16 ± 4	0.3 ± 0.3	414 ± 31	7 ± 4	2.2 ± 0.5	1.2 ± 0.1

Note: SD; standard deviation (n = 3).

lignocellulosic ethanol index (LEI) = (xlyan + glucan)/lignin.

torrefaction index (TI) = lignin/(xlyan + arabinan + mannan + galactan).

## Discussion

Measuring the mass fraction of non-structural carbohydrates and chlorophyll fluorescence parameters, we reveal that mature bamboos (especially age 3 bamboos) probably provide carbohydrates to young bamboos (age 1) for rebuilding photosynthetic systems in winter, which confirms the behaviour of mature bamboos to feed offspring for maintaining the survival probability of young individuals. Seasonal changes and plant ages are important factors influencing the mass fraction of NSCs^12^. Plants can adjust their carbon budgets to meet their physiological demands in their life cycle^14^. There is an important relationship between the adaptation of carbohydrates in different seasons and physiological changes in plants. The mass fraction of carbohydrates is not unchanged and will be affected by various environmental and climatic factors^15^.

Photosynthesis, a biochemical reaction in which plants produce carbohydrates, supplies the energy and carbon sources needed for various metabolic functions^16^, and converts approximately 100–115 tG C per year on Earth^17^. Although photosynthesis occurs between different kinds of plant, chloroplasts are still the main place for photosynthesis, which are divided into light and light-independent reactions, in most different plants. Light reaction converts light energy into chemical energy (ATP and NADPH) via an electron transport chain^18^. Through the light-independent reaction, the energy obtained by the light reaction is consumed for RuBisCO to synthesise carbohydrates which provide the metabolic and biosynthetic pathways required in plants^19^. Photosynthesis is processing in chloroplasts, in which light reactions are carried out on the thylakoid membrane of the chloroplast, which has a plurality of photosystems and protein complexes. In all the photosystems and protein complexes, photosystem II (PSII) is the most sensitive to light reaction, and it is suitable as an indicator for measuring photosynthesis ability of plants^20^. When the PSII is functional in light reaction, the amount of fluorescence emitted by the chloroplast is pretty low. However, in some conditions, the light reaction may synthesise too much energy. If these energies are not consumed, reactive oxygen species (ROSs) will be produced^21^. ROSs will first destroy the chloroplast membrane and thylakoid membrane, which will impair photosynthesis function and increase PSII maximum quantum yield (Fv/Fm)^20^. Thereby, ROSs also destroy unsaturated fatty acids in plants^22^, deoxyribonucleic acid (DNA), carbohydrates^23^, and proteins^24^. The above reasons cause abnormal metabolism and synthesise in plant cells. For describing the phenomenon that mature bamboos feed carbohydrates to young bamboos, the mass fraction of NSCs and chlorophyll fluorescence parameters in different bamboo ages and seasonal changes will be discussed later. Following this, for multiple usage purposes, the mass fraction of SCs will reveal suit timing and ages to harvest bamboos.

Moso bamboos’ NSCs (such as soluble sugars and starches) and some parts of SCs are like the total storage of available energy for plants. When plants growing, for instance, to form SCs (such as cellulose, hemicellulose, and lignin), to synthesise some anti-stress proteins, or to maintain self-energy consumption, they must be consumed NSCs. When plants consume more soluble sugars, the starch stored in each part is hydrolysed into oligosaccharides, which are further hydrolysed into soluble monosaccharides or disaccharides for metabolic or synthetic use^10,14^. When old bamboos are dying (≥5 age) or the canopy of bamboo forest is closed, carbon sources will be completely redistributed^25^. Some SCs hydrolyse into disaccharides, and then into monosaccharides. After that, for making other individuals easier to survive, old bamboos synthesise sucrose which may be transported to other young or mature bamboos by rhizome systems.

The sucrose and starch at the bottom of young bamboo (age 1) in spring have a fairly high mass fraction (Figs. 2, 3, and 4). Some studies suggest that the nutrients and NCs demand of growing bamboo shoots may be transported from mature bamboos via rhizome systems^5^. Other studies have pointed out that mass fraction of sucrose is higher when the transport amount of NCs is large^9,26,27^. In this study, the leaf function of juvenile bamboo was not fully developed, and the photosynthesis ability was not yet completed (Fig. 6). Therefore, young bamboos need a lot of NCs transported from mature bamboos via rhizome systems and redistributed carbohydrates for young bamboos which accumulate a large amount of starch at the bottom. So that young bamboos can grow and develop without a self-contained carbon source.

In summer, because the photosynthesis systems of mature bamboos (age 2, 3, and 4) is functional (Figs 6–9), the ability to produce carbohydrates is workable as well. Almost all mass fraction of NSCs is low no matter in young or mature bamboos because they are used to transfer into SCs and to other biosynthesis and metabolism.

When the autumn comes, bamboos will begin to store starch in order to survive during the winter, so the mass fraction of starch gradually increase. It is worth noting that at this time, the TSCs (Fig. 2) and sucrose (Fig. 4) at the bottom of young bamboos are higher than middle and top in autumn. Fv/Fm (Fig. 6) and Φ value (Fig. 8) fall in October. The mass fraction of sucrose in the bottom, middle and top of mature bamboos (age 2, 3, and 4) is obviously low (Fig. 4). The reason may be that the photosynthetic ability of young bamboos is destroyed by the first wave of cold current in October. We also calculated the net photosynthetic rate in leaves of age 1 bamboo and respiration rate in age 1 bamboos using environmental data, nonlinear regression models and parameters of chlorophyll fluorescence (PCF)-net photosynthetic rate (NPR) models approach^1,2,28–32^. The conducted results reveal that the net photosynthetic rate (7.15 kgCO_2_ ha^−1^ day^−1^) is lower than the respiration rate (8.55 kgCO_2_ ha^−1^ day^−1^) of age 1 bamboos in October. On the other hand, other mature bamboos are still health, the photosynthesis systems are still workable, and the carbohydrates are synthesised as usual. For young bamboos surviving during the winter, those carbohydrates synthesised from mature bamboos are transporting to the bottom of young bamboos in the form of sucrose. Autumn also enters the beginning of the dry season in Taiwan, soluble sugar plays an important role in plant osmotic regulation^33^, when the plant faces water stress, the sucrose/starch ratio increases^34^. When entering the fall, the sucrose/starch ratio increased in the age 1, 2, and 4 bamboos (Fig. 5).

When winter comes, Moso bamboos have begun to consume the starch accumulated in the late summer and early autumn. At this time, a large amount of starches is in the bottom of age 3 bamboos (Fig. 3), and mass fraction of TSCs (Fig. 2) and sucrose (Fig. 4) are also in a high level. It is speculated that age 3 bamboos are the highest productive among the whole bamboo forests. Therefore, it may still produce carbohydrates and continuously synthesise starches during the winter solstice, and it will continue to decompose and transport to other young bamboos. Old bamboos (age ≥ 5), due to their aging stage, have almost no soluble carbohydrates (Figs 2, 4), indicating that their energy consumption and flow is low (Table 1). The transferable SCs have been diverted because of the low lignocellulosic ethanol index (LEI). In winter, due to the lack of precipitation and low temperature, the ratio of sucrose/starch in different ages of bamboos is relatively higher than in other seasons. In this condition, plants tend to decompose starch, increase the mass fraction of soluble carbohydrates, and act with calcium ions to stabilise the cell membrane and prevent cell leakage^35^ or to regulate infiltration^33^.

Based on the above, mature bamboos may probably transfer sucrose to young bamboos if they encounter low temperature stress, so that they can survive in the cold winter. However, old bamboos have a low transport capacity of sucrose and is less productive than mature bamboos.

On the other side, through the measurement of chlorophyll fluorescence, we can determine the photosynthetic system capacity of bamboos and understand the process of the PSII, electron transferring in light reaction, ATP synthesis, and even CO_2_ fixation, and then can directly or indirectly observe the entire photosynthesis. Parameters are selected as follows:

Maximum quantum yield (Fv/Fm) is the maximum light efficiency of PSII under dark adaptation^36,37^ and the most commonly used determinant of photosynthesis efficacy. Fv/Fm, the best indicator of photoinhibition^37^, is almost a constant (0.832 ± 0.004) for healthy plants. However, Fv/Fm decrease when plants’ leaves damage. In this study, Fv/Fm was between 0.7 and 0.8. However, in April and October, the young bamboo leaves (age 1) showed a significant decrease (Fig. 6). The reason is that new bamboo leaves are developing in April and their photosynthetic systems have not yet fully developed. Furthermore, in October, due to the first wave of cold current attacked, young bamboos’ photosynthetic systems and thylakoids are damaged by low temperature stress. Above reasons probably lead Fv/Fm decrease.

Minimum fluorescence (Fo) means that fluorescence is measured under the circumstance which PSII in the dark-adapted state are opening, all QA are in oxidation state^38^, and qN (non-photochemical quenching) is in the smallest condition. Using the PAM instrument, Fo can be measured by measuring radiation (MR; single light between 665–685 nm; < 0.2 μmole m^−2^ s^−1^). When plants under stress, Fo often rises. This phenomenon indicates that the rate of excitation energy entering the P680 reaction centre is decreased in PSII, or part of the chlorophyll antenna is disconnected from P680^39^. In this study, Fo of bamboo leaf is between 0.40–0.55. However, age 1 bamboo leaves have a significant increase in winter and May (Fig. 7). It may be as described above; the young bamboo leaves are under attack by cold current. Some chlorophyll antennas are separated from the reaction centre of PSII because young bamboos (age 1) are not fully developed in May. Chloroplasts may contain many precursors of light-harvesting complex bind protein (LHCB), which cause Fo in young bamboo leaves is higher than matures (age 2, 3, and 4).

Quantum yield (Φ), that is, the light quantum yield of PSII under light adaptation, or the efficiency of electron transfer after PSII absorbs a photon^20^. The Φ of Moso bamboo leaves in this study is around 0.5–0.7. However, there is a significant decrease in young bamboo leaves between autumn and winter (especially in October when the first wave of cold current strikes) (Fig. 8). The result is the same as Fv/Fm.

Photochemical quenching (qP) means that the photochemical capacity and the number of QA oxidation states in PSII under light adaptation^40^.

$${\rm{qP}}=({\rm{Fm}}^{\prime} -{\rm{Fs}})/({\rm{Fm}}^{\prime} -{\rm{Fo}}^{\prime} )$$

When oxygen evolution is harmed, the electrons transferred to QA from P680 decrease and qP rises. Otherwise, if the process after PSII (such as PQ movement, PSI, Pc electron transfer, and ATP synthesis, even more CO^2^ fixation) does not pass well, electrons at QA do not pass to the next step and qP finally decreases. The qP value of this study is often between 0.7–0.9. When the age 4 bamboos grow new leaves in November, the qP value is obviously low. When the age ≥ 5 bamboo leaves are in July, the qP value is larger than other ages (Fig. 9). This shows that the part of mature bamboos (especially in age 4) which are turning into old bamboos (age ≥ 5) has low qP because the photosynthesis is not going well, even if the new leaves are grown and PSIIs are functional. The oxygen evolution in old bamboo leaves has been hurt, so the qP is therefore higher than other ages of bamboo leaves.

Above evidences indicate that physiological functions of the photosynthesis in bamboo leaves are functional in all seasons. However, young bamboo leaves temporarily are malfunctioning due to the cold damage [Through PCF-NPR models approach, the result indicates that the net photosynthetic rate (7.15 kgCO_2_ ha^−1^ day^−1^) is lower than the respiration rate (8.55 kgCO_2_ ha^−1^ day^−1^) of age 1 bamboos in October]. Following this, those young bamboo leaves probably get some NSCs from mature bamboos and rebuild their photosynthetic systems gradually. As for the old bamboo leaves, the subsequent electron transfer of the photosynthesis system is blocked, the efficiency of producing carbohydrates is slow, and the physiological functions are gradually going downward.

The results also showed that the average mass fraction of lignin was 30.5%, xylan was about 23.9%, arabinan was 1.7%, mannan was 0.3%, glucan was 42.0%, and galactan was about 0.7% in Moso bamboos. For evaluating the best felling and utilization timing of Moso bamboo, we created 2 indexes for each applied strategy in this study:

- lignocellulosic ethanol index (LEI) = (xlyan + glucan)/lignin
- torrefaction index (TI) = lignin/(xlyan + arabinan + mannan + galactan)

The higher LEI is, the better the yield of lignocellulosic ethanol and pulp. Similarly, if TI is high, the yield and quality of the torrefying will increase as well. The average LEI is about 2.2 during a year and higher in spring than other seasons. The LEI is highest among age 4 bamboos in spring and age 1 bamboos in summer; it is lowest among age ≥ 5 bamboos in summer, autumn, and winter and age 4 bamboos in autumn. Glucans are usually composed of cellulose, partially hemicellulose, starch, and soluble sugars^41^. In spring, the age 1 bamboo is not fully developed (Fig. 4), and receives the carbohydrates from mature bamboos (age 2, 3, and 4). Therefore, the mass fraction of starch is higher than that of other seasons and bamboo ages. Xylan, arabinan, galactan, and mannan are the major constituent sugar groups of hemicellulose^42^. When bamboo is used, it will be pyrolysed over 275 °C. Therefore, the higher the mass fraction of hemicellulose is, the lower the yield and quality of torrefaction will be^43^.

Lignin is a high molecular polymer composed of oxyphenylpropanol-based monomers filled with cellulose, hemicellulose and pectin^24^. The higher the mass fraction of lignin is, the less yield of the pulp and paper production will be. And it causes the yield of lignocellulosic ethanol decrease; furthermore, the cost of acid hydrolysis increase. Therefore, the higher the lignin mass fraction is, the more unfavourable for pulping and papermaking and cellulosic ethanol is as well. However, lignin’s’ properties are relatively stable and less susceptible to pyrolysis. When the mass fraction of lignin is high, the yield and quality of torrefaction increase.

Spring is the beginning of the growing season, bamboos will first tend to synthesise cellulose and hemicellulose, so the average LEI [it means (xylan + glucan)/lignin ratio] is higher than other seasons. The more glucan compose in bamboo, the higher yield of producing lignocellulosic ethanol and pulp will be^44^. The age 1 bamboos are the main carbon sink in Moso bamboo forests. Therefore, mature bamboos transport the remaining carbohydrates to them. The dry matter accumulates very quickly. In spring, a large amount of starch is stored and used to grow it till the summer. Hence, there is a high level of LEI in young bamboos which suit for producing lignocellulosic ethanol and pulp especially in summer. Before entering aging (age 5 bamboos), age 4 bamboos have the highest accumulation of dry matter and a higher level of LEI. However, they still do not apply to product lignocellulosic ethanol because they enter aging stage in the fall and occur in the condition which redistribute cellulose hydrolysis and transfer the remaining carbohydrates to other immature bamboos, and then the LEI is significantly decreased. After that winter, they become into old bamboos (age ≥ 5).

The results show that young bamboos (age 1) in summer or old bamboos (age ≥ 5) (Fig. 3) can reduce the chance of bamboo products being infected by insects. In summer, the age 1 bamboos with a smaller breast diameter can be harvested as a raw material for biomass energy and pulp & paper (Table 1). Harvesting old bamboo (age ≥ 5) in summer, autumn, and winter can be used for bamboo torrefaction or bamboo vinegar production (Table 1). Thus, the age 1 bamboos in summer had the higher LEI, suited to produce lignocellulosic ethanol. And the age 4 bamboos in spring had the higher LEI, either, it still did not suit for producing lignocellulosic ethanol, which was caused by its higher production of soluble carbohydrates (Fig. 1).

The age 5 bamboos, which suited to apply torrefying of biocoal production, were the higher TI in the season of summer, autumn and winter. Due to it would fall into aging and might occur cellulose hydrolysis redistribution, which transferred to other young bamboo and rhizome systems that needed to use carbohydrates^13^. Therefore, the TI increased in age 5 bamboos. Consolidated NSC (Figs 1, 2, and 3) and structural carbohydrates (Table 1) in Moso bamboos showed that age 5 bamboos were supplied for handicraft production, due to the lowest starch content (Fig. 2) that was a high correlation with the resistance to insects^45–47^. The age 1 Moso bamboos were the highest LEI in summer, therefore, we could harvest the small DBH age 1 bamboo in summer for the usage of lignocellulosic ethanol, pulp and paper, and could leave the large one to increase the total site productivity^6^. Biocoal production used the age 5 bamboos, which were lower TI (Table 1) in summer, autumn and winter, due to low hemicelluloses and high lignin content which was increasing the yield and the quality of torrefying^43^.

## Conclusion

After cold stress attacked, carbohydrates demand was higher than supply in young Moso bamboos. During this period, almost all the NSCs mass fraction of the bottoms in mature bamboos were re-allocated and transferred to the young bamboos via underground rhizomes for both the rebuilt photosystems and the metabolism of the young bamboos. The transfer process of NSCs from attached mature bamboos to immature bamboos perhaps stopped when the leaves of the young bamboos could re-provide enough photoassimilates to meet the demand of carbohydrates for the young bamboos. This phenomenon elaborated as a kind of lactation behaviour in the society of Moso bamboos.

## Materials and Methods

### Samples collection

All Moso bamboo samples were obtained during 2011 in a Moso bamboo forest (24°04′27~29″N; 121°00′24~58″E) which located in Huisun Forest Area, Experimental Forest Management Office, National Chung Hsing University at Nantou County, Taiwan. These bamboo samples were manually collected 15 cm long from the bottom, middle, and top by using a hand saw, frozen by liquid nitrogen in −196 °C (77 K), loaded in a small fridge, and transported back to the lab afterwards. After that, they were microwaved for 1 min and dried at 70 °C for 1 day before analysing carbohydrates.

### Analysing carbohydrates in bamboo culm

In order to analyse carbohydrates of bamboo samples, the bamboo culms were grinded and sieved to the maximum particle size of 100 mesh (particle size ≦ 0.149 mm) by a high-speed grinding machine. Then, samples were placed in fridge below −20 °C until the analyses were carried out. Their mass fraction of carbohydrates and lignin were determined according to described methods previously in technical report of determination of structural carbohydrates and lignin in biomass provided by National Renewable Energy Laboratory^48–50^.

#### Non-structural carbohydrates - Total soluble sugars (TSS)

For all the experimental operates, around 0.10 g of sample was used. A text tube added deionised water (DI Water) 10 cm^3^, vortexed well, and then put it in water bath at 65 °C for 2 hours^48^. Following this, it centrifuged 3,018.6 ×g for 13 min. We pipetted supernatant 5 cm^3^ from the text tube into a volumetric flask which added DI water in it until 100 cm^3^. The TSS solution was prepared. After that, prepared another test tube and pipetted the TSS solution 0.1 cm^3^, added DI Water 1.9 cm^3^, 9% phenol 0.1 cm^3^, and 96% H_2_SO_4 (aq)_ 6 cm^3^ into it, and then vortexed it for 15 seconds. Placed the text tube for 30 min, measured its absorbance at a wavelength of 485 nm by a spectrophotometer (Double Beam UV–VIS, Thermo, USA), and made a standard curve for getting the concentration of it as well. Equation (1) illustrated the percentage of TTS (%) in samples:

1 $${\rm{TTS}}\,{\rm{or}}\,{\rm{Starch}}( \% )={\rm{Conc}}.({\rm{\mu }}{\rm{mole}}\,{{\rm{cm}}}^{-3})\times {\rm{Dilute}}\times (10/{\rm{Weight}})\times (180/10,000)$$

Conc. = the concentration of TTS or starch in sample

Dilute = the dilution of sample solutions

Weight = the weight of samples

#### Non-structural carbohydrates - Starch

Following above, we collected precipitate into another text tube, added DI water 5 cm^3^, centrifuged them 3,018.6 ×g for 13 min, and then removed supernatant^49^. Precipitates ovend at 65 °C for 16 hr. Following this, the text tubes contained precipitates were added DI water 2 cm^3^, and then put them into water bath at 100 °C for 15 min. After that, added 9.2 N HClO_4 (aq)_ 2 cm^3^ in each tube, vortexed for 15 seconds, added DI water 6 cm^3^, vortexed for 15 seconds again, and then centrifuged 3,018.6 xg for 13 min. We prepared another test tube, pipetted the supernatant 0.1 cm^3^, added DI water 1.9 cm^3^, 9% phenol 0.1 cm^3^ and 96% H_2_SO_4 (aq)_ 6 cm^3^, vortexed for 15 seconds, and waited for 30 min. We measured the supernatant’s absorbance at a wavelength of 485 nm by the spectrophotometer, and made a standard curve for getting the concentration of it as well. Equation (1) illustrated the percentage of starch (%) in samples.

#### Non-structural carbohydrates - Analysis of soluble carbohydrates

This method covered the determination of carbohydrates, expressed as the percent of each sugar present in a hydrolysed sample. The supernatant which prepared in above for TSS was syringed 0.5 cm^3^ and injected through filter (0.45 μm) into a column (CarboPacTM PA1, Dionex, USA) of a liquid chromatography machine (ICS-3000, Dionex, USA). The analysis of the soluble carbohydrates composition (e.g. glucose, fructose, sucrose) was performed.

#### Structural carbohydrates and lignin

In the first part of SCs and lignin’s process, the samples placed in a crucible, recorded weight and then put in an ashing furnace up to 575 ± 25 °C for 1 hour^50^. After cooling it in the oven, recorded its weight again and prepared it for the following analysis.

#### Structural carbohydrates and lignin – Acid insoluble lignin (AIL)

Grabbed each sample 0.3 g into each 125 ml conical flask, added 72% H_2_SO_4 (aq)_ 3 cm^3^, stirred by a glass stirring rod, and put all conical flasks into water bath at 30 ± 3 °C for 60 ± 5 min^50^. After that, added 84 ± 0.04 cm^3^ DI water into each conical flask.

After above steps completed, washed the 0.45 μm filters twice, placed in a crucible, dried at 105 °C in a drying cabinet, and then cooled and recorded the weight of them. Following this, the liquid, which was in conical flask, stirred well by a glass stirring rod again, filtered by a method of vacuum decompression, and conserved at −20 °C for the determination of acid soluble lignin (ASL) and SCs. Once above steps had finished, the residue on the filter washed by DI water several times, put in a crucible which was dried at 105 °C in a drying cabinet until the weight unchanged, and then cooled and recorded the weight of them. Following this, placed them into an ashing furnace, was heating at 105 ± 25 °C for 12 min, then turning up to 250 ± 25 °C for 30 min, turning up again to 575 ± 25 °C for 180 min, and then turning down to 105 ± 25 °C for 6 hr. Took out the crucible from the ashing furnace to the drying cabinet, cooled and recorded their weights as well. Equation (2) illustrated the percentage of AIL (%) in samples:

2 $${\rm{AIL}}( \% )=({{\rm{W}}}_{({\rm{crucible}}+{\rm{residue}}+{\rm{filter}})})-{{\rm{W}}}_{({\rm{crucible}}+{\rm{ash}})}-{{\rm{W}}}_{({\rm{filter}})}/{\rm{W}}({\rm{sample}})$$

W_(crucible+residue+filter)_ = crucible + residue + filter (g)

W_(crucible+ash)_ = crucible + ash (g)

W_(filter)_ = filter (g)

W_(sample)_ = sample (g)

#### Structural carbohydrates and lignin – Acid soluble lignin (ASL)

The filtrate, which was from above, was measured the absorbance at a wavelength of 198 nm by the spectrophotometer, and made 4% H_2_SO_4 (aq)_ solution for blanks. Equation (3) illustrated the percentage of ASL (%) in samples.

3 $${\rm{ASL}}( \% )=({{\rm{UV}}}_{({\rm{abs}})}\times {{\rm{V}}}_{({\rm{filtrate}})}\times {\rm{D}}/{\rm{\varepsilon }}\times {{\rm{W}}}_{({\rm{sample}})}\times {{\rm{L}}}_{({\rm{pathlength}})})\times 100$$

UV_(abs)_ = absorbance

V_(filtrate)_ = the volume of filtrate (l)

D = the dilution of sample solutions

ε = extinction coefficient L g^−1^ cm^−1^ (25 in this study)

W_(sample)_ = sample (mg)

L_(pathlength)_ = the length of UV-Vis cell (cm) (nearly 1 cm in this study)

#### Structural carbohydrates and lignin – Total lignin (TA)

The sum of ASL and AIL is the sum of TA.

#### Analysis of structural carbohydrates

The liquid, which came from above, was pipetted 1 cm^3^ to a test tube, then added 0.05 g Ca (CO_3_)_2(S)_ until pH 7, topped up H_2_O _(l)_ till 10 cm^3^, and then vortexed for 10 sec and placed for 15 min. After the solution was clarified, injected 0.5 cm^3^ into a 0.45 μm filter head which was binding a column (CarboPac^TM^ PA1, Dionex, USA) and an ion chromatography system (ICS-3000, Dionex, USA) for analysing the components of structural carbohydrates which was including glucan, xylan, arabinan, mannan, and galactan.

### Data processing and statistical analysis Method for measuring the ability of photosynthetic systems (photosystems)

For measuring the ability of bamboo leaves’ photosystems, we selected 3 to 5 samples of each bamboo age (1, 2, 3, 4, and ≥5) in the Moso bamboo forest every month from July 2011 to June 2012. Specific aluminium leaf clamps (Leaf-Clip Holder 2030-B) were used and the leaf samples were selected for dark adaptation for 30 minutes. And then used the portable chlorophyll fluorometer, which would provide the measuring light (2 μmol photon m^−2^ s^−2^) and saturating light (850 μm photon m^−2^ s^−2^), to determine the efficiency of primary conversion of light energy of photosystems (Fv/Fm), and record data by Data Acquisition Software, DA-2000).

### Statistical analysis

Data presented in this paper were the average of 3 replicates. A one-way analysis of variance (ANOVA) was conducted to test the different seasons and ages effects on the non-structural carbohydrates, structural carbohydrates and lignin. When the ANOVA indicated a significant treatment effect, the least significant difference (LSD) test was used to recognize means in different ages or seasons. And a level of 0.05 for significance was used in all statistical analysis, in which were performed using SAS 9.2 (SAS Institute, Cary, NC, USA).
